# Musical Pitch Perception and Categorization in Listeners with No Musical Training Experience: Insights from Mandarin-Speaking Non-Musicians

**DOI:** 10.3390/bs15010030

**Published:** 2024-12-31

**Authors:** Jie Liang, Fen Zhang, Wenshu Liu, Zilong Li, Keke Yu, Yi Ding, Ruiming Wang

**Affiliations:** 1 School of Music, South China Normal University, Guangzhou 510006, China; 2021010105@m.scnu.edu.cn; 2 Philosophy and Social Science Laboratory of Reading and Development in Children and Adolescents, Ministry of Education, & Center for Studies of Psychological Application, School of Psychology, South China Normal University, Guangzhou 510631, China; 2023023826@m.scnu.edu.cn (W.L.); 2021010238@m.scnu.edu.cn (Z.L.); wangrm@scnu.edu.cn (R.W.); 3 LIBIS, KU Leuven, 3001 Heverlee, Belgium; fen.zhang@kuleuven.be

**Keywords:** musical pitch, pitch perception, pitch discrimination, categorical perception

## Abstract

Pitch is a fundamental element in music. While most previous studies on musical pitch have focused on musicians, our understanding of musical pitch perception in non-musicians is still limited. This study aimed to explore how Mandarin-speaking listeners who did not receive musical training perceive and categorize musical pitch. Two experiments were conducted in the study. In Experiment 1, participants were asked to discriminate musical tone pairs with different intervals. The results showed that the nearer apart the tones were, the more difficult it was to distinguish. Among adjacent note pairs at major 2nd pitch distance, the A4–B4 pair was perceived as the easiest to differentiate, while the C4–D4 pair was found to be the most difficult. In Experiment 2, participants completed a tone discrimination and identification task with the C4–D4 and A4–B4 musical tone continua as stimuli. The results revealed that the C4–D4 tone continuum elicited stronger categorical perception than the A4–B4 continuum, although the C4–D4 pair was previously found to be more difficult to distinguish in Experiment 1, suggesting a complex interaction between pitch perception and categorization processing. Together, these two experiments revealed the cognitive mechanism underlying musical pitch perception in ordinary populations and provided insights into future musical pitch training strategies.

## 1. Introduction

Music is a complex auditory experience composed of fundamental elements such as pitch, speed, strength, and timbre. Among them, pitch—an auditory sensation produced by variations associated with musical melody or, more fundamentally, the perceptual correlate of the frequency of a sound waveform—plays a key role in a variety of auditory and musical processes, including music appreciation, speech intonation, and auditory scene analysis (Deutsch et al., 2004; Moore, 2008). In the everyday listening environment, pitch-evoking sounds are usually complex tones, composed of a series of harmonic components whose frequencies are integer multiples of fundamental frequency (F0) (Plack et al., 2014). The variations in pitch between notes form the patterns necessary for the structure of music. Previous studies have primarily focused on musical pitch perception in musicians who possess refined pitch recognition skills, while fewer have examined these abilities in the general population (McClaskey, 2017; McDermott et al., 2010; Sares et al., 2018). Understanding the cognitive foundations of these processes has important implications for music education and could help refine teaching strategies aiming at improving pitch-related tasks.

In studies of musical pitch perception, researchers have focused primarily on pitch discrimination, particularly the ability to distinguish between musical tones at different intervals. In general, the farther the tones are apart (i.e., the greater the interval differences), the easier it is to distinguish the pitches. Musicians who have received extensive aural training show greater accuracy and sensitivity as the difference in intervals increases (Zarate et al., 2012) and low discrimination thresholds (McClaskey, 2017). Compared with musicians, non-musicians performed worse in discriminating musical tone pairs and were less sensitive to musical pitch variations (McClaskey, 2017; McDermott et al., 2010; Sares et al., 2018). These studies suggest that pitch-interval discrimination is influenced by the size of the intervals being compared and by musical training. However, these studies did not consider listeners’ tonal language experience. Many studies suggested that tonal language experience contributes to musical tone perception (Asaridou & McQueen, 2013; Bidelman et al., 2011). Non-musicians with tonal language experience may perform similarly as musicians on pitch-interval discrimination (Chang et al., 2016). 

The pitch interval varies between different notes. Within an octave, the pitch interval between two notes consists of the 2nd (minor and major), 3rd (minor and major), 4th (perfect and augmented), 5th (perfect), 6th (minor and major), 7th (minor and major), 8th (perfect). Most previous studies examined pitch intervals in certain degrees but did not systematically examine all the seven types of intervals. For example, McClaskey (2017) tested the 2nd (minor), 4th (augmented), and 5th (perfect), and McDermott et al. (2010) tested 1, 1.5, 2, 2.5, and 3 semitones. A systematic examination on different degrees of pitch intervals could contribute to better revealing musical pitch discrimination patterns. For listeners who have not received long-term musical training but whose native language is a tonal language, whether musical pitch discrimination is related to notes and pitch intervals remains to be explored. 

In music processing, melodic information is considered to be represented in two ways. One is contour coding that describes the pattern of pitch changes and the other is interval coding that represents the pitch distance between successive notes (Fujioka et al., 2004). Understanding how humans process these elements is vital not only for advanced musical representations, but also for pitch discrimination and music perception. A key aspect of pitch perception that parallels speech processing is categorical perception. Originating from speech perception research, categorical perception refers to the brain’s ability to group continuous acoustic stimuli into discrete categories, often influenced by language experience (Diehl et al., 2004; Möttönen & Watkins, 2009). That is, when listeners hear continuously varying pitch stimuli, they tend to perceive these stimuli as belonging to distinct pitch categories. It has been commonly tested using discrimination tasks (Baker & Osgood, 1954), in which small increments between pitch stimuli are presented in a random order and listeners must judge whether two stimuli are the same or different, and using identification tasks (Miyazaki, 1988), in which participants are asked to label a tone as belonging to a specific musical note. Generally, the discriminability is higher for cross-category than within-category stimuli pairs, even if the degree of physical differences is the same. The categorization does not change linearly with the physical differences. Instead, it changes drastically to the opposite category around the perceptual boundary. Pitch plays a crucial role in distinguishing between phonemes and conveying meaning (i.e., lexical tones) in tonal languages. Research has shown that lexical tone perception processes follow categorical perception principles (Xi et al., 2010). Listeners display distinct category boundaries and discrimination peaks when processing pitch variations in lexical tones (Yu et al., 2014). 

In recent years, categorical perception has also been discussed in the field of musical pitch (Chang et al., 2016; Turner et al., 2019). Categorical perception is not limited to speech but also extends to non-speech sounds like music, suggesting that common mechanisms exist for pitch perception across different auditory domains (Turner et al., 2019). In both music and language, categorical perception relies on the ability to organize sounds into meaningful units, although the features used by the brain in each domain may differ. Music primarily uses pitch, while language relies on phonological elements such as phonemes (J. Harris & Lindsey, 1995). Early studies indicated that musicians display a categorical perception of intervals, whereas untrained listeners do not display natural categorization (Burns & Ward, 1978). Although early studies did not explicitly report whether musician participants have tonal language experience or not, later studies found that native speakers of tonal languages can perceive phonological categories through pitch, as well as pick up subtle acoustic differences in music, cues for cross-domain pitch perception (A. Chen et al., 2016). There may be shared mechanisms between music and language in how these categories are formed and maintained (Bradley, 2018).

Although non-musicians possess the ability to categorize musical pitch, this ability is weaker compared to that of musicians (e.g., Chang et al., 2016; S. Chen et al., 2020; Weidema et al., 2016). Experienced-based bidirectional transfer of pitch proficiency between speech and music has been observed in previous studies. For example, musical training enhanced the categorization of Mandarin lexical tones, with English-speaking musicians recognizing lexical tones similar to their native Mandarin-speaking patterns. English-speaking musicians also showed higher categorization ability than English-speaking non-musicians. Furthermore, the experience of Mandarin lexical tones influenced melodic pitch recognition, with Mandarin-speaking non-musicians approximating English-speaking musicians and showing more categorization patterns compared to English-speaking non-musicians (Chang et al., 2016). However, it remains unclear to what extent the categorization perception of Mandarin-speaking non-musicians is. Moreover, in non-musicians with tonal language as native language, if discriminating musical pitch is related to notes and pitch intervals, then do different categorical perception features cause their different performance on pitch interval discrimination? The issue also remains to be investigated. 

This study aims to further investigate the cognitive processing involved in musical pitch perception and categorical perception by conducting two experiments. Specifically, we focus on two questions: How well do non-musicians who speak tonal languages discriminate musical pitch, and is this discrimination ability affected by pitch intervals? Another question is whether there are differences in categorical perception for different continuous pitches in non-musicians? Our experiments were conducted to explore the above questions. In Experiment 1, we adopted a tone discrimination task to investigate how Mandarin speakers without formal musical training experience discriminate between musical tone pairs with distinct pitch distances. We hypothesized that the nearer apart the pitches were, the more difficult it was to distinguish. In Experiment 2, we further adopted a categorical perception paradigm with tone identification and discrimination tasks to explore how they categorize musical pitch. We hypothesized that the participants’ responses to the two tasks would follow the pattern of categorical perception. However, the extent of categorization would be varied between different tone pairs.

## 2. Experiment 1

In this experiment, we explored the perceptual discrimination for musical tone pairs at different distances in Mandarin speakers without formal music training experience.

### 2.1. Method

#### 2.1.1. Participants

Thirty university students from South China Normal University with Mandarin as their native language (16 females and 14 males; age = 21.40 ± 2.42) completed the experiment. According to self-report and pre-experimental questionnaires in the lab, all participants had not received professional music training except for the required music courses in school. The questionnaires included the following items: (1) Did you receive any formal music (vocal and instrumental) training during college?; (2) Did you receive general music classes during college?; (3) Did you receive any formal music (vocal and instrumental) training before college?; (4) Did you receive general music classes before college?; (5) Can you sing in tune?; (6) Can you tell when others are off-key? Additionally, all participants were right-handed, with normal vision or corrected vision, normal hearing, and verbally reported no neurological or mental illness. This study was conducted in accordance with the principles outlined in the Declaration of Helsinki. Ethical approval was obtained from the Ethics Committee of South China Normal University. All participants provided written informed consent before participating in the study and received 20 RMB as compensation after the experiment.

#### 2.1.2. Materials

The musical pitch stimuli were derived from the eight fundamental pitches between C4 and C5 of a piano, including C4 (262.29 Hz), D4 (294.63 Hz), E4 (330.87 Hz), F4 (350.54 Hz), G4 (392.98 Hz), A4 (441.51 Hz), B4 (495.55 Hz), and C5 (524.42 Hz). These were generated using Cubase 10.5 software (Steinberg Media Technologies GmbH, Hamburg, Germany) with the timbre set to a grand piano (Galaxy Vienna Grand Library). The stimuli were sampled at a frequency of 44,100 Hz with a 16-bit depth and a bit rate of 705 kbps. Each stimulus was standardized to a duration of 600 ms and an intensity of 70 dB SPL using the software Praat (version 6.4.23) (Boersma & Weenink, 2024) before being incorporated into the formal experiment.

With these stimuli, we constructed 8 types of same-tone pairs and 28 types of different-tone pairs. The same-tone pairs included C4–C4, D4–D4, E4–E4, F4–F4, G4–G4, A4–A4, B4–B4, and C5–C5. The different-tone pairs included C4–D4, C4–E4, C4–F4, C4–G4, C4–A4, C4–B4, C4–C5, D4–E4, D4–F4, D4–G4, D4–A4, D4–B4, D4–C5, E4–F4, E4–G4, E4–A4, E4–B4, E4–C5, F4–G4, F4–A4, F4–B4, F4–C5, G4–A4, G4–B4, G4–C5, A4–B4, A4–C5, and B4–C5. According to the pitch interval, these different-tone pairs were divided into eight categories: 2nd, 3rd, 4th, 5th, 6th, 7th, and 8th. Table 1 shows the detailed arrangements.

#### 2.1.3. Procedure

**Figure 1:**
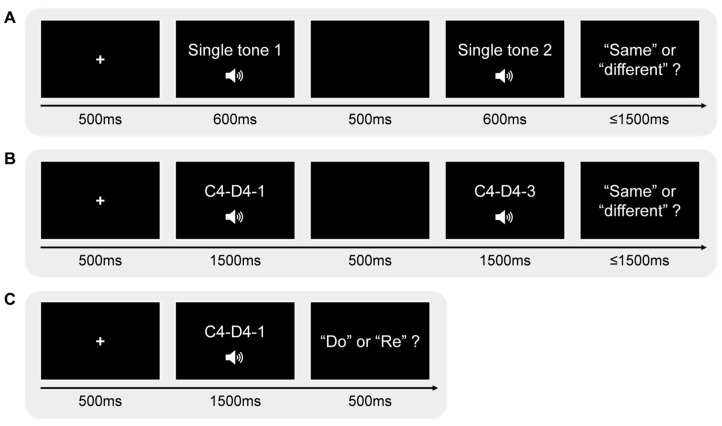
Experimental procedure. (**A**) Discrimination task in Experiment 1. Participants were instructed to discriminate whether two consecutive presented stimuli were the same or different. (**B**) Discrimination task in Experiment 2. Participants discriminated whether two consecutive presented stimuli from a musical tone continuum were the same or different. (**C**) Identification task in Experiment 2. Participants identified the solfege names of the presented stimuli of a musical tone continuum.

The experiment consisted of two phases: a practice phase and a formal experiment. The practice phase included 12 trials (same-tone pairs: 6 trials; different-tone pairs: 6 trials), while the formal experiment comprised 112 trials (same-tone pairs: 56 trials; different-tone pairs: 56 trials). The practice trials consisted of F#4 (371.27 Hz), G#4 (416.72 Hz), and A#4 (467.77 Hz), which we did not use in the formal experiment. They were a fixed set of trials that stayed the same for all participants. In the formal experiment, the same-tone pairs were repeated 7 times, and the different-tone pairs were repeated twice. The same-tone and different-tone pairs were presented randomly to each participant. The sequence of two notes in each different-tone pair was counterbalanced. For example, the C4–D4 pair consisted of a C4–D4 trial and a D4–C4 trial. Participants took a break midway through the formal experiment. Each trial began with a fixation point (“+”) displayed at the center of the screen for 500 ms. Following this, a single tone lasting 600 ms was played, followed by a second tone of the same duration, with a 500 ms interval between the two tones. After hearing the second tone, participants judged whether the two tones were the same in 1500 ms, pressing the “F” key for the same tones and the “J” key for different tones. The participants did not receive feedback in the practice or the formal experiment. The assignment of key presses was counterbalanced across participants (for half of the participants, “F” = same and “J” = different, but for the other half, “F” = different and “J” = same). After pressing the button, the experiment entered the next trial. The entire experiment took approximately 15 minutes. The experimental program was created using E-Prime 3.0 (Psychology Software Tools, 2021). The experimental procedure is outlined in Figure 1A. 

#### 2.1.4. Data Analysis

We focused on participants’ performance on the different-tone pairs. The accuracy and response time for the same-tone pairs are shown in Table 2. In the data processing, the non-response trials were excluded (4.5% of the data) because these data may affect the findings’ accuracy and effectiveness. The accuracy refers to whether the participants respond to the tone pairs correctly, with same-tone pairs being “yes” while different-tone pairs being “no”. The response time was recorded when the participants pressed the button after the onset of the second tone in a trial. We calculated each participant’s mean accuracy and reaction time in each condition and then entered the data into the statistical analyses. The response time was subjected to natural logarithm transformation before entering the analysis. A one-factor repeated-measures ANOVA was used, with the pitch distance of different-tone pairs (the 2nd, 3rd, 4th, 5th, 6th, 7th, and 8th) as the independent variable and accuracy and response time as the dependent variables. We also calculated the Pearson correlation coefficients between pitch distance and accuracy and between pitch distance and response time, using the integers 2, 3, …, and 8 to represent the 2nd, 3rd, …, and 8th intervals. Moreover, we conducted regression analyses with pitch distance as the independent factor and reaction time and accuracy as the dependent factor. There were 210 datapoints in these analyses, respectively. 

### 2.2. Results

#### 2.2.1. Pitch Distance

**Figure 2:**
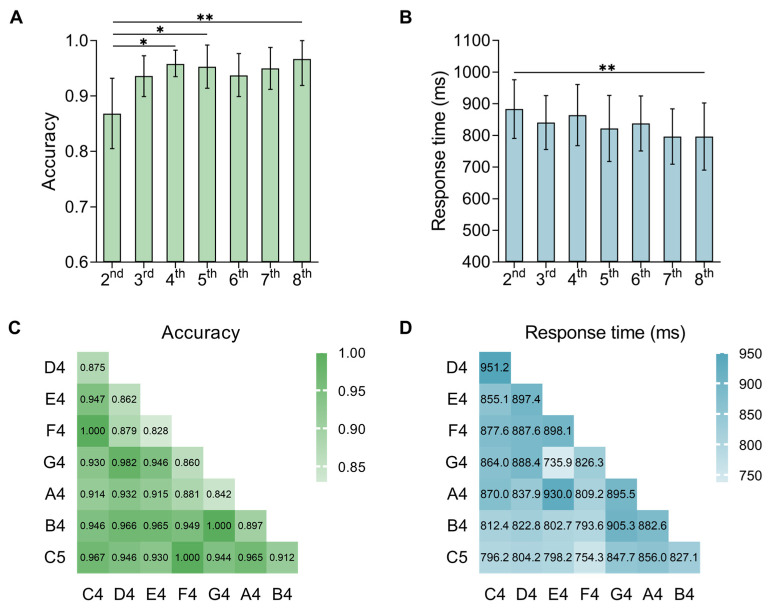
Accuracy and response time for different-tone pairs at different distances. (**A**) Accuracy of different-tone pairs at different distances. (**B**) Response time of different-tone pairs at different distances. (**C**) Accuracy of different-tone pairs at 2nd distances. (**D**) Response time of different-tone pairs at 2nd distances. * *p* < 0.05, ** *p* < 0.01. Error bars represent a 95% confidence interval.

The average accuracy and response time of different-tone pairs with different pitch distances are shown in Figure 2A,B and Table 2. The results revealed a significant main effect related to the accuracy (*F*(6, 174) =3.000, *p* = 0.028, $\eta_{p}^{2}$ = 0.320) and the response time (*F*(6, 174) = 3.231, *p* = 0.015, $\eta_{p}^{2}$ = 0.462) of pitch distance. Regarding the accuracy, the post hoc test with the FDR correction method showed that the different-tone pair with 2nd pitch distance was significantly lower than that with 4th (*p* = 0.019), 5th (*p* = 0.039), and 8th (*p* = 0.007) pitch distance. The accuracy of the different-tone pair with 2nd was marginally significantly lower than that of the different-tone pair with 7th (*p* = 0.059). There was no significant difference between the other pitch distances (*p* > 0.1). Regarding the response time, the post hoc test with the FDR correction method showed that the pitch distance of 2nd was significantly higher than that of the 8th (*p* = 0.007) and marginally higher than that of the 7th (*p* = 0.059). The other pitch distances showed no significant difference between each other (*ps* > 0.1). 

In addition, we found that pitch distance was significantly positively correlated with accuracy (*r*(210) = 0.186, *p* = 0.007) and was negatively correlated with response time (*r*(210) = −0.133, *p* = 0.053), though the correlation was weak. The regression analyses showed that pitch distance significantly positively predicted accuracy (*β* = 0.011, 95%CI = [0.001,0.02], *R*^2^ = 0.035, *t* = 2.728, *p* = 0.007) and marginal significantly negatively predicted reaction time (*β* = −0.019, 95%CI = [−0.04, 0.00], *R*^2^ = 0.018, *t* = −1.94, *p* = 0.053). 

#### 2.2.2. Relatively Easy and Difficult Different-Tone Pairs at 2nd Pitch Distance

**Figure 3:**
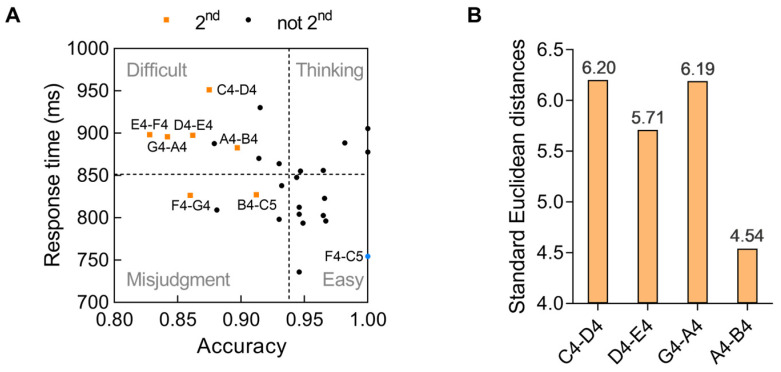
(**A**) The relative positions of different-tone pairs at the 2nd and other distances (not 2nd) mapped to the two-dimensional space. The intersection is the median of the accuracy (93.862%) and the response time (850.265 ms). F4–C5 is also marked as it was used as the reference to calculate the standard Euclidean distances. (**B**) The standard Euclidean distances between the 2nd different-tone pairs and F4–C5.

As revealed by the ANOVA, correlation, and regression analyses on pitch distance, the different-tone pairs at the 2nd pitch distance were the most difficult pairs to discriminate. However, these tone pairs consisted of minor 2nd and major 2nd pairs. We then conducted exploratory analyses on these pairs’ data. The accuracy and response times for each specific different-tone pair at this distance are shown in Figure 2C,D. We map each different-tone pair at the 2nd distance and other distance into a two-dimensional space, with the horizontal axis representing the accuracy rate and the vertical axis representing the response time. Median is a common way to differentiate individuals’ performances. If participants perform longer reaction times and lower accuracy than the median on a certain pair, the pair is difficult to discriminate. However, if participants perform shorter reaction times and higher accuracy than the median on a certain pair, the pair is easy to discriminate. Based on the logic, with the intersection of the median of the accuracy (93.862%) and the response time (850.265 ms) as the origin, the entire two-dimensional space is divided into four quadrants (Figure 3A). 

The first quadrant in the upper right corner is “thinking”. In this quadrant, the participants used a longer time to obtain a higher accuracy rate. The participants can obtain the correct answer after careful consideration. The second quadrant in the lower right corner is “easy”. In this quadrant, the participants used a shorter time to obtain a higher accuracy rate. The participants do not need to think for a long time and can obtain the correct answer. The third quadrant in the lower left corner is “misjudgment”, that is, the participants used less time to obtain a lower accuracy rate. This shows that when faced with these tone pairs, the participants are prone to misjudgment and are more likely to decisively choose the wrong answer. The fourth quadrant in the upper left corner is “difficult”, and the participants took longer on the response time but obtained a lower accuracy rate. This shows that it is very difficult to distinguish these tone pairs and even if the participants spent more cognitive resources, they did not obtain a higher accuracy rate.

To find out the relatively easy and difficult tone pairs at the 2nd pitch distance, we first excluded the pairs at the minor 2nd (E4–F4, B4–C5). This exclusion was made because the pitch differences between the major and minor 2nd are not equivalent and could influence the outcomes of the research. Considering that the F4–G4 pair was located in the “misjudgment” area, we also excluded this pair. Then, we identified the easiest and most difficult pair from the rest of the four pairs (C4–D4, D4–E4, G4–A4, A4–B4) at the major 2nd. We calculated the standard Euclidean distance from the four pairs to the pair that participants responded to most fast and accurately (F4–C5, reaction time: 753.563 ms, accuracy: 100%). As shown in Figure 3B, the distance between C4–D4 and F4–C5 (6.20) was the largest, suggesting that C4–D4 was the most difficult tone pair among the four pairs. The distance between A4–B4 and F4–C5 (4.54) was the smallest, suggesting that A4–B4 was the easiest pair among the four pairs.

### 2.3. Discussion

Combining the ANOVA, correlation, and regression analyses, we found that the greater the pitch distance between two pairs of tones, the better the participants perform in the discrimination task. This effect is similar to that in musicians who can easily tell the difference between distant tones but generally have lower difference thresholds for discrimination tasks compared to non-musicians (Bianchi et al., 2016; Sares et al., 2018). Moreover, the different-tone pairs at the 2nd pitch distance were the most difficult pair to discriminate. We further identified the relatively easy and difficult tone pairs at the 2nd pitch distance. According to the standard Euclidean distances, among C4–D4, D4–E4, G4–A4, and A4–B4 pairs, we identified the A4–B4 pair as the easiest to distinguish, while the C4–D4 pair was the most challenging. There were two potential explanations for the difference between the C4–D4 and A4–B4 pairs. One is that the C4–D4 pair and A4–B4 pair come from different frequency ranges (200–300 Hz vs. 400–500 Hz). Previous studies indicated that pitch discrimination is dependent on frequency range (J. D. Harris, 1952; McPherson & McDermott, 2018). So, the different frequency ranges may result in the difference. Another is the categorical perception mechanism underlying these two pairs. Listeners may exploit different patterns to discriminate between C4 and D4 and A4 and B4. In Experiment 2, we further explore whether there is a categorical perception pattern in the perception of the two tone continua, C4–D4 and A4–B4. We hypothesized that there were differences in the categorical perception of these two different-tone pairs, which may be a potential reason for their different discrimination results in Experiment 1. 

## 3. Experiment 2

In Experiment 2, we used tone pairs A4–B4 and C4–D4 as experimental materials to explore the categorical perception of the musical pitch continuum.

### 3.1. Method

#### 3.1.1. Participants

Sixty participants were recruited in Experiment 2. A total of 30 participants (16 females and 14 males; age = 19.83 ± 1.66) were randomly assigned to the C4–D4 tone continuum stimulation condition. The other 30 participants (12 females and 18 males; age = 22.80 ± 2.64) were in the A4–B4 tone continuum stimulation condition. The participants also completed the pre-experimental questionnaires as Experiment 1 in the lab. All participants were native Mandarin speakers from South China Normal University with no professional music training, right-handed and with normal or corrected vision, normal hearing, and no neurological or psychiatric disorders. This study was conducted in accordance with the principles outlined in the Declaration of Helsinki. Ethical approval was obtained from the Ethics Committee of South China Normal University. All the participants did not take part in Experiment 1. They provided written informed consent before participating in the study and received 25 RMB as compensation after the experiment.

#### 3.1.2. Materials

**Figure 4:**
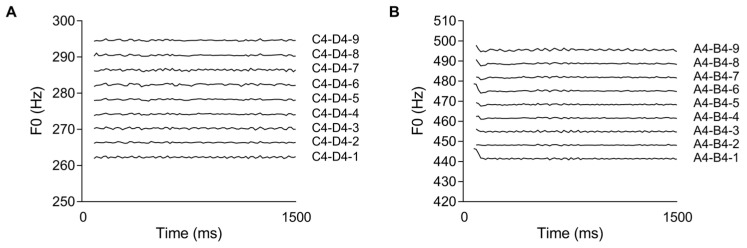
The fundamental frequency (F0) for the pitch features of each stimulus in the C4–D4 (**A**) and A4–B4 (**B**) continua.

We used the C4–D4 and A4–B4 tone pairs to generate the C4–D4 and A4–B4 tone continua with Cubase 10.5 software (Steinberg Media Technologies GmbH, Hamburg, Germany). Specifically, the C4 and D4 stimuli were used as the endpoint to create a C4–D4 continuum consisting of nine stimuli (C4–D4-1/C4–D4-2/C4–D4-3/C4–D4-4/C4–D4-5/C4–D4-6/C4–D4-7/C4–D4-8/C4–D4-9). The A4 and B4 were used as the endpoint to create an A4–B4 continuum consisting of nine stimuli (A4–B4-1/A4–B4-2/A4–B4-3/A4–B4-4/A4–B4-5/A4–B4-6/A4–B4-7/A4–B4-8/A4–B4-9). These stimuli were generated automatically at the same time when we used C4 and D4 (A4 and B4) as the endpoint to create the C4–D4 (A4–B4) tone continuum in the software. The sampling frequency of the pitch stimulus was 44100 Hz, and the sampling bit was 16 bit and 705 kbps. The timbre was changed to the grand piano (Galaxy Vienna Grand Library) timbre before output. The software Praat (version 6.4.23) (Boersma & Weenink, 2024) was used for standardization of duration (1500 ms) and intensity (70 dB SPL) before being used in the formal experiment. When synthesizing stimuli, the units of the starting pitch of the stimulus are converted from Hertz (Hz) to the equivalent rectangular bandwidth (ERB), which is a psychoacoustic standard that can truly reflect the actual perceived frequency changes (Greenwood, 1961). The stimuli of the two tone continua are shown in Figure 4. Table 3 presents the pitch parameters of the stimuli start and end points of the two tone continua.

#### 3.1.3. Procedure

We adopted the categorical perception paradigm (Peng et al., 2010; Xi et al., 2010) in Experiment 2. The paradigm consisted of two tasks: discrimination and identification tasks. The experimental procedures of the two tasks are shown in Figure 1B,C, respectively. In total, 30 participants were stimulated by the C4–D4 tone continuum, and 30 participants were stimulated by the A4–B4 tone continuum. In the discrimination task, participants were required to judge whether the two sounds they heard were the same. If they were the same, they would press the “F” key, and if they were different, they would press the “J” key. The keys were balanced among the participants. The participants heard two stimuli one after another. The experimental stimuli were divided into the same-stimulus pairs (C4–D4-1-1/C4–D4-3-3/C4–D4-5-5/C4–D4-7-7/C4–D4-9-9) and different-stimulus pairs (C4–D4-1-3/C4–D4-3-1/C4–D4-3-5/C4–D4-5-3/C4–D4-5-7/C4–D4-7-5/C4–D4-7-9/C4–D4-9-7). The different-stimulus pairs were referred to as D13 (C4–D4-1-3/C4–D4-3-1), D35 (C4–D4-3-5/C4–D4-5-3), D57 (C4–D4-5-7/C4–D4-7-5), D79 (C4–D4-7-9/C4–D4-9-7) in the following. The same-stimulus pairs were presented 16 times, and the different-stimulus pairs were presented 10 times. All stimulus pairs were presented randomly, resulting in a total of 160 trials. Each stimulus lasted 1500 ms, with an interval of 500 ms between stimuli. The entire discrimination task lasted about 25 minutes. The procedure for the A4–B4 tone continuum discrimination task was the same as that for the C4–D4 tone continuum.

In the identification task, the participants were required to identify the tone names (solfege) of stimuli, for example, to identify the sound of the stimulus as ”do” (C4) or ”re” (D4), press the ”F” key for “do”, and press the ”J” key for “re”, and the key presses were balanced among the participants. It is noted that we used solfege instead of pitch notation in the task by considering that non-musician listeners are generally familiar with solfege because they were taught in required music lessons in primary and secondary schools. The 9 stimuli in the tone continuum appeared 20 times each, resulting in a total of 180 trials. Each stimulus was presented for 1500 ms. When the participant responded by pressing a key, the next trial would be presented. The task procedure of the A4–B4 tone continuum was the same as that of the C4–D4 tone continuum. The experiment was presented using E-prime 3.0 (Psychology Software Tools, 2021). The entire identification task lasted about 20 min.

#### 3.1.4. Data Analysis

In the discrimination task, we conducted a two-factor mixed-measure ANOVA, with the independent variables being the tone continuum and the stimulus pair. The tone continuum was a two-level between-group variable (C4–D4/A4–B4), and the stimulus pair was a four-level within-group variable (D13/D35/D57/D79). The dependent variable was the discrimination score, P (e.g., Peng et al., 2010). The discrimination score, P, was calculated as shown in the following equation:

$$P=P\left(\frac{SS}{S}\right)*P\left(S\right)+P\left(\frac{DD}{D}\right)*P(D)$$

where P(SS/S) represents the proportion of same-stimuli pairs judged as same, and P(S) represents the proportion of same-stimuli pairs to all stimulus pairs; P(DD/D) represents the proportion of different-stimuli pairs judged as different, and P(D) represents the proportion of different-stimuli pairs to all stimulus pairs. Taking D13 in the tone continuum C4–D4 as an example, the probability of matching judgments for C4–D4-1-1 and C4–D4-3-3 pairs, P(SS/S), is calculated by dividing the sum of same judgments for both pairs by the total number of trials for these pairs. The overall probability, P(S), is the total number of C4–D4-1-1 and C4–D4-3-3 trials over all possible C4-D4 trials. For different judgments, P(DD/D), it is the ratio of different judgments for C4–D4-1-3 and C4–D4-3-1 to the total trials of these pairs, while P(D) considers the proportion of different trials.

In the identification task, we conducted a one-factor non-repeated measures ANOVA. The independent variable was the tone continuum, which was a two-level between-group variable (C4-D4/A4-B4). The dependent variables were the category boundary position, the category boundary width, and the identification curve slope, respectively. We calculated whether each subject judges the tone of each stimulus to be “do” (C4) or ”re” (D4) and made a recognition curve for each subject. Then, probit analysis was performed on the identification curve (Hallé et al., 2004; Peng et al., 2010). That is, the data at 50% is defined as the category boundary, and the linear distance between 25% and 75% is defined as the category width, and the regression coefficient, *β*, of regression lengthening is defined as the slope of the identification curve. We calculated the participants’ category boundary position, category boundary width, and identification curve slope under different tone continua through the identification curve. SPSS 26.0 was used to perform statistical analysis on the data, corrected for post hoc comparisons using the Bonferroni method.

### 3.2. Results

#### 3.2.1. Discrimination Task

**Figure 5:**
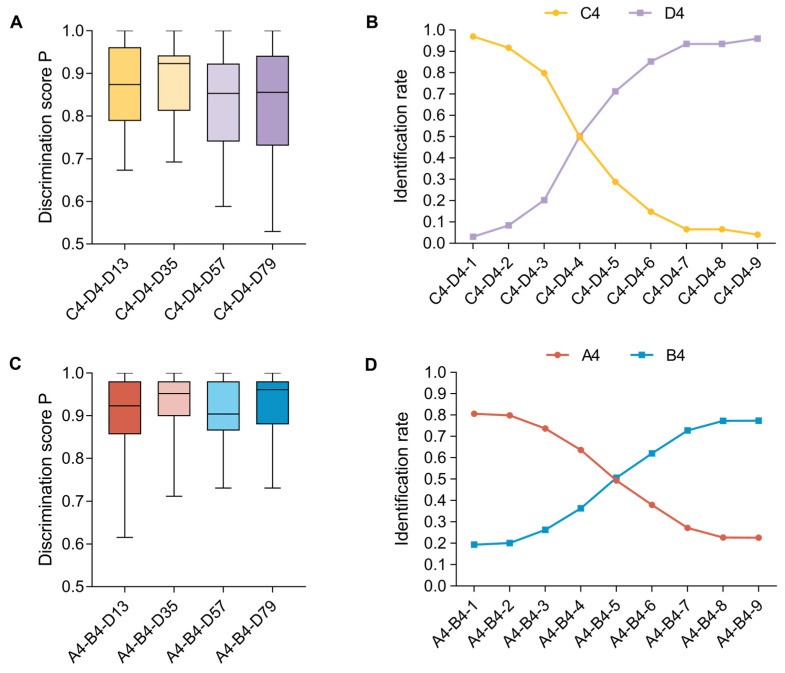
The results of the discrimination and identification task in Experiment 2. (**A**) Discrimination score P of C4–D4 tone continuum. (**B**) Identification rate of C4–D4 tone continuum. (**C**) Discrimination score P of A4–B4 tone continuum. (**D**) Identification rate of A4–B4 tone continuum.

The results of the discrimination task of the two tone continua are shown in Figure 5A,C and Table 4. We found that the interaction between the tone continuum and the stimulus pair was significant (*F*(3, 174) = 4.118, *p* = 0.007, $\eta_{p}^{2}$ = 0.066). The simple effect test showed that under the C4–D4 continuum, the discrimination score P of D35 was significantly greater than that of D57 (*p* < 0.001) and D79 (*p* = 0.29). There was no significant difference between other pairs (*p* > 0.1). No significant difference was found between all stimulus pairs under the A4–B4 continuum (*p* > 0.1). 

The main effect of tone continuum was significant (*F*(1, 58) = 8.277, *p* = 0.006, $\eta_{p}^{2}$ = 0.125). Post hoc tests showed that the discrimination score P of the C4–D4 tone continuum was significantly lower than that of the A4–B4 tone continuum. The main effect of stimulus pair was significant (*F*(3, 174) = 4.118, *p* = 0.007, $\eta_{p}^{2}$ = 0.066). Post hoc tests showed that the discrimination score P of D35 was significantly higher than that of stimulus pair D57 (*p* < 0.001), and there was no significant difference between the other stimulus pairs (*p* > 0.1).

#### 3.2.2. Identification Task

The identification curves of the two tone continua are shown in Figure 5B,D. The descriptive statistics of the category boundary position, category boundary width, and identification curve slope of the two tone continua are shown in Table 4. In the category boundary position, there was a significant difference between the two tone continua (*F*(1, 58) = 4.578, *p* = 0.037, $\eta_{p}^{2}$ = 0.073), and the post hoc test showed that the category boundary position of the C4–D4 tone continuum was significantly smaller than that of the A4–B4 tone continuum (*p* = 0.037). In the category boundary width, there was no significant difference between the two tone continua (*F*(1, 58) = 2.083, *p* = 0.1.54, $\eta_{p}^{2}$ = 0.035). In the slope of the identification curve, there was a significant difference between the two tone continua (*F*(1, 58) = 18.264, *p* < 0.001, $\eta_{p}^{2}$ = 0.239). Post hoc tests showed that the slope of the identification curve of the C4–D4 tone continuum was significantly smaller than that of the A4–B4 tone continuum (*p* < 0.001).

### 3.3. Discussion

In Experiment 2, we first found that the discrimination score of D35 was significantly greater than that of D57 and D79 under the C4–D4 continuum but not under the A4–B4 continuum. Regarding the position of the category boundary reflected by the identification results (shown in Figure 5B,D), we found that the boundaries of A4–B4 and C4–D4 are both located between A4–B4-4 and A4–B4-5 and C4–D4-4 and C4–D4-5. The findings suggested that the stimuli before the boundary tended to be perceived as A4/C4, while the stimuli after the boundary tended to be perceived as B4/D4. Considering the discrimination of stimulus pairs near this boundary, especially D35, where A4–B4-3 and A4–B4-5, and C4–D4-3 and C4–D4-5 belong to different categories, this discrimination should be better than the discrimination of other stimulus pairs if the category effect is strong. However, we only found the difference under the C4–D4 continuum, but not under the A4–B4 continuum, indicating that the category effect of C4–D4 is stronger. In addition, the slope of C4–D4 is steeper than that of the A4–B4 tone continuum (shown in Figure 5B,D), and the boundary width of C4–D4 is smaller, which also indicates that its category effect is stronger than that of A4–B4 continuum. Compared to earlier studies that found untrained listeners lacked natural categorical perception, unlike musicians (Burns & Ward, 1978), the results suggested that Mandarin-speaking listeners do exhibit categorical perception. However, the C4–D4 tone continuum demonstrated a stronger capacity for categorical perception compared to the A4–B4 continuum.

## 4. General Discussion

Two experiments were conducted in the current study to explore how Mandarin non-musicians who had not received professional music training perceive musical pitch. Results of Experiment 1 showed different responses to discriminate between musical tone pairs with different pitch intervals. The correlation and regression analyses further suggested that the greater the distance between the two tones, the better the participants performed in the discrimination task. We also identified that among the tone pairs with major 2nd pitch distance (C4–D4, D4–E4, G4–A4, and A4–B4), the easiest to distinguish was A4–B4, and the hardest to distinguish was C4–D4, suggesting that the perceived difficulty varies even if the pitch interval is the same (one whole tone). Experiment 2 further indicated that Mandarin non-musicians showed stronger categorical perception ability to the C4–D4 tone continuum compared with the A4–B4. The two experiments suggested specific musical pitch perception mechanisms in Mandarin non-musicians who had not received professional music training.

Results of Experiment 1 indicated that musical tone pairs at the 2nd pitch distance in an octave were the most difficult for Mandarin non-musician listeners to discriminate. Recognizing the differences between neighboring note pairs is essential for musical perception and performance, involving identifying subtle variations in pitch, rhythm, and timbre. Trained musicians can accurately distinguish pairs of identical notes with different tunings. In contrast, non-musicians perform at a chance level, especially in harmonic situations (simultaneous notes) compared to melodic situations (successive notes) (Mathews & Sims, 1981). However, we found that Mandarin-speaking listeners can perceive those neighboring tone pairs differently, with A4–B4, C4–D4, D4–E4, E4–F4, and G4–A4 were considered as difficult, while F4–G4 and B4–C5 were easily misjudged. The notes at different frequency ranges and categorical perceptions underlying different notes may explain the findings.

Using the identified tone pairs at the major 2nd pitch distances that were easiest (A4–B4) and hardest (C4–D4) to distinguish, we found that the C4–D4 tone continuum was more effectively classified, with steeper slopes and smaller recognition curves than for the A4–B4 tone continuum. This result may explain the results of Experiment 1, where the C4–D4 pair had slower response times and lower accuracy, and the A4–B4 pair showed faster response times but similarly lower accuracy. Listeners may tend to identify C4–D4 pairs on the basis of learned categories (e.g., the role of the notes in the scale), which rely on the knowledge of music theory or the typical structure of a scale to distinguish these notes. In contrast, the A4–B4 pair may be perceived more based on their physical acoustic properties (e.g., pitch or timbre) rather than the theoretical or categorical roles of the notes. However, it is unknown whether the difference between the two pairs comes from the tones themselves or different degrees of familiarity with the solfege. The non-musician listeners may be more familiar with the solfege “do” and “re”, compared to “la” and “si”, so they are able to rely more on musical knowledge and structure when the “do–re” solfege is used, regardless of the actual acoustic stimuli. Therefore, we need to be cautious of the findings in Experiment 2. 

Pitch perception in music is a complex phenomenon that intertwines with categorical perception. Our findings, combined with our previous research on lexical tone processing (Yu et al., 2019) provide further evidence that an individual’s ability to perceive pitch in speech and music is closely related, but also varies. This is consistent with previous research on speech and musical pitch, which found shared cognitive processing (Perrachione et al., 2013). However, such categorical perception in music may differ across the domains for different pitch pairs. This result suggests that the mechanism of categorical perception is different in the domains of music and language. As in lexical tone perception, the stronger the categorical perception, the better the tone differentiation effect (Bidelman & Lee, 2015), while it may not always be the case with musical pitch perception. This is consistent with a study that investigated whether the mechanisms controlling pitch contour perception differ from speech and music and demonstrated domain-specific cognitive mechanisms with top-down effects (Weidema et al., 2016). 

The present study focused on the non-musicians who have tonal language experience. Findings from the present study could provide implications for future studies and music training. Firstly, we identified musical tone pairs with different perceptual difficulties. Further studies could investigate how various factors, such as language background (e.g., tonal or non-tonal language), music training, and the general auditory processing mechanism, interact to influence the perception of musical note pairs. Secondly, considering non-musicians’ limited musical experience, the non-musician listeners may be more familiar with the solfege “do” and “re” compared to “la” and “si”. Future studies could compare the “do–re” and “la–si” solfeges with “C4–D4” and “A4–B4” in categorical perception tasks. The comparison could contribute to indicating whether the notes’ names modulate categorical perception of musical pitch. Lastly, we revealed different difficulties in musical tone pairs at different pitch distances. Future studies could consider how to promote improving the difficult musical tone pairs (e.g., F4–G4 and B4–C5) in music training. For example, listeners could use hand gestures to simulate these musical tones’ pitch features. Previous studies have indicated the role of hand gestures in lexical tone learning (Yu et al., 2024). It may also function in musical tone training. 

It is worth noting that there are several limitations of this study. The first was the relatively small sample size (30 participants in Experiment 1 and 30 participants per tone continuum in Experiment 2). Future studies should recruit more participants. Secondly, in Experiment 2, we assigned participants to one of two conditions to prevent participants from feeling tired, which may affect their performance on the task. However, it may be a limitation of the experimental design. It would be better to use a within-subject design in the experiment by eliminating the potential effect of individual differences. Thirdly, the two experiments lacked the measure of participants’ auditory working memory. Future studies should consider the individual differences in this aspect. Lastly, we recruited participants from a non-musical population with a specific tonal language (Mandarin) background. It lacks comparison with listeners with other language and music backgrounds. Further research could investigate the differences in musical pitch perception among tonal and non-tonal language speakers with minimum formal musical training experience, and non-tonal-speaking musicians.

## 5. Conclusions

Our results show that the closer the pitch distance of musical tones, the more challenging they are to discriminate. The C4–D4 tone continuum may elicit stronger categorization effects compared to the A4–B4 continuum, despite C4–D4 being the most difficult to differentiate, and A4–B4 was the easiest one among adjacent note pairs at the major 2nd pitch distance. Overall, these two experiments demonstrate the complex interplay between musical pitch perception and categorization processes and shed light on the cognitive mechanisms involved in music processing by non-musicians who are native speakers of a tonal language. The findings also provided implications for musical training. 

## Figures and Tables

**Table 1:** The different-tone pairs and their corresponding pitch interval categories.

Pitch Interval		Different-Tone Pairs
2nd	Minor 2nd	E4–F4, B4–C5
	Major 2nd	C4–D4, D4–E4, F4–G4, G4–A4, A4–B4
3rd	Minor 3rd	D4–F4, E4–G4, A4–C5
	Major 3rd	C4-E4, F4–A4, G4–B4,
4th	Perfect 4th	C4–F4, D4–G4, E4–A4, G4–C5
	Augmented 4th	F4–B4
5th	Perfect 5th	C4–G4, D4–A4, E4–B4, F4–C5
6th	Minor 6th	E4–C5
	Major 6th	C4–A4, D4–B4
7th	Minor 7th	D4–C5
	Major 7th	C4–B4
8th	Perfect 8th	C4–C5

**Table 2:** Accuracy and response times of different-tone pairs with different pitch distances and same-tone pairs in Experiment 1.

		Accuracy		Response Time (ms)	
Different-Tone Pairs	Pitch Distance	*M*	*SD*	*M*	*SD*
	2nd	0.868	0.170	883.304	248.088
	3rd	0.936	0.098	840.708	228.347
	4th	0.958	0.065	864.198	258.882
	5th	0.953	0.104	822.044	280.255
	6th	0.937	0.104	837.981	232.807
	7th	0.950	0.102	796.356	234.635
	8th	0.967	0.127	796.217	284.412
Same-tone pairs	Tone				
	C4	0.924	0.265	770.970	270.594
	D4	0.958	0.200	784.922	279.297
	E4	0.933	0.250	805.092	312.788
	F4	0.961	0.195	778.488	293.549
	G4	0.932	0.253	752.408	256.183
	A4	0.938	0.243	781.057	283.989
	B4	0.964	0.186	765.685	271.750
	C5	0.974	0.161	728.810	235.873

**Table 3:** Pitch parameters of the stimuli in the C4–D4 and A4–B4 continua.

Tone Continuum	Starting Pitch (Hz)	Starting Pitch (ERB)	Step (Hz)	Step (ERB)
C4–D4				
9	294.63	7.19	4.14	0.08
8	290.49	7.11	4.07	0.07
7	286.42	7.04	4.06	0.07
6	282.36	6.97	4.13	0.08
5	278.23	6.89	4.06	0.07
4	274.17	6.82	3.90	0.07
3	270.27	6.75	3.92	0.08
2	266.35	6.67	4.06	0.07
1	262.29	6.60		
A4–B4				
9	495.55	10.23	6.86	0.09
8	488.69	10.14	6.82	0.09
7	481.87	10.05	6.69	0.09
6	475.18	9.96	6.83	0.09
5	468.35	9.87	6.75	0.09
4	461.60	9.78	6.67	0.09
3	454.93	9.69	6.76	0.10
2	448.17	9.59	6.66	0.09
1	441.51	9.50		

Note. ERB = Equivalent rectangular bandwidth.

**Table 4:** Descriptive statistics of the two tone continua under different stimulus pairs, including category boundary position, category boundary width, and identification curve slope.

Tone Continuum	D13	D35	D57	D79	Category Boundary Position	Category Boundary Width	Identification Curve Slope
C4-D4	0.87 ± 0.10	0.88 ± 0.08	0.83 ± 0.11	0.84 ± 0.12	4.25 ± 0.68	2.51 ± 1.83	−0.73 ± 0.34
A4-B4	0.90 ± 0.09	0.93 ± 0.07	0.91 ± 0.08	0.92 ± 0.08	4.77 ± 1.14	0.82 ± 6.15	−0.36 ± 0.33

## Data Availability

The data presented in this study are available on request from the corresponding authors due to their containing information that could compromise the privacy of the research participants.
